# Factors that influence the complications and outcomes of femoral neck fractures treated by cannulated screw fixation

**DOI:** 10.1038/s41598-020-57696-2

**Published:** 2020-01-20

**Authors:** Nikolai Ramadanov, Ionel Toma, Harald Herkner, Roman Klein, Wilhelm Behringer, Gerrit Matthes

**Affiliations:** 1 Center for Emergency Medicine, University Hospital Jena, Friedrich Schiller University, Am Klinikum 1, 07747 Jena, Germany; 2 Clinic for Reconstruction and Trauma Surgery, Ernst von Bergmann Hospital, Charlottenstr. 72, 14467 Potsdam, Germany; 3 University Clinic for Emergency Medicine, Medical University Vienna, Währinger, Gürtel 18-20, 1090 Wien, Austria; 4 Orthopaedics, Trauma Surgery and Sports Traumatology, Marienhaus Hospital, Hetzelstift, Stiftstr. 10, 67434 Neustadt, Germany

**Keywords:** Medical research, Outcomes research

## Abstract

To investigate the influence of various factors on the two outcome parameters “procedure - specific complication” (femoral head necrosis, infection, nonunion, femoral neck shortening, screw loosening, implant penetration) and “functional outcome” in patients with displaced and undisplaced femoral neck fracture treated by cannulated screw fixation. All cases of a femoral neck fracture, operated by cannulated screw fixation, in the period from December 2014 to December 2017 were included. The observation period of the included patients was 12 months. Information on their outcome was collected after evaluation of current x-ray images and on request from the responsible further treatment physician. Continuous data were presented as mean value ± standard deviation, categorical data as absolute and relative frequency. The effect of potential factors on endpoints was estimated with a multivariable logistic regression analysis and 95% confidence intervals calculated. The null hypothesis Odds Ratio = 1 was checked by the Wald test. The likelihood ratio test was used to test for deviation from linearity. The mean age of the 56 included patients was 72 years (36 min, 96 max), 44.5% (n = 25) were male and 55.5% (n = 25) female. The femoral neck fractures were classified as follows: Garden I: 73%, Garden II: 16%, Garden III: 11%, Pauwels I: 73%, Pauwels II: 21%, Pauwels III: 5%, 31-B1: 73%, 31-B2: 27%, 31-B3: 0%. The factor patient age showed a statistically significant influence on the outcome parameter procedure-specific complication. None of the remaining factors examined showed a statistically significant influence on both outcome parameters procedure-specific complication and functional outcome. 69% of the patients from age 80 onwards suffered a procedure-specific complication. A rate of 41% procedure-specific complications as an outcome parameter in trauma surgery shows a necessity for improvement. The increasing risk of procedure-specific complications for patients with a femoral neck fracture treated by cannulated screw fixation is associated with rising patient age. A more stable head-perserving operative method or an endoprosthetic procedure should be considered in high-risk patients (≥80 y.o.).

## Introduction

The number of hip fractures worldwide is expected to increase to 2.6 million in 2025 and 4.5 million in 2050^1^. The treatment of femoral neck fractures gives the surgeon almost a free decision on the surgical procedure within the scope of applicable guidelines. The choice is between endoprosthetic and femoral head preserving methods^2^. However, the benefit of maintaining the femoral head can be associated with numerous complications: femoral head necrosis, nonunion, femoral neck shortening, screw loosening, implant penetration^2^. Numerous studies on the outcome of head-preserving femoral neck fractures have a relatively high rate of osteosynthesis failure, in some cases >40%^3–7^. Some studies about non-displaced femoral neck fractures with head-preserving operation have shown that higher age is associated with an increase in revisions, complications, nonunion and poor functional outcome^8–10^. Other studies have shown that age is not associated with earlier mortality, osteosynthesis failure, or functional outcome^11–14^. However, the factors affecting the outcome of displaced and undisplaced femoral neck fractures treated by a head-preserving operative method have not been adequately studied. The subject of this study is the displaced and undisplaced femoral neck fracture treated by cannulated screw fixation. The aim was to investigate the influence of various factors on the two outcome parameters “procedure - specific complication” and “functional outcome”.

## Methods

### Data collection

All cases of a femoral neck fracture, operated by cannulated screw fixation, in the period from December 2014 to December 2017 were included. The investigated factors were taken from the hospital information system Cerner Soarian Clinicals Version 4.1. In the process, surgical reports, discharge letters and quality assurance data sheets were evaluated. The observation period of the included patients by our retrospective study was up to 12 months. Information on their outcome was collected after evaluation of current x-ray images in the hospital information system and on request from the responsible further treatment physician. The follow-up time was 12 months. 5 patients, who died during the follow-up time, were excluded from the study.

### Classification of fractures

The included fracture types were classified by two experienced trauma surgeons independently (κ = 0.96) on admission X-ray images according to the classification of Garden and Pauwels. In divergent cases, a third trauma surgeon helped make the right decision.

### Determination of procedure - specific complication and functional outcome

The short-term and long-term complications after cannulated screw fixation of femoral neck fractures (femoral head necrosis, infection, nonunion, femoral neck shortening, screw loosening, implant penetration) were summarized as “procedure - specific complication” (PSC). The PSC was determined after a systematic evaluation of X-ray images 12 months postoperatively. The functional outcome was determined as equal or worse by a systematic comparison of the preoperative walking distance and the walking distance in the 12th postoperative month. A five-stage scoring system was used according to possible walking distance: unlimited walking distance, walking distance up to 500 m, walking distance up to 50 m, mobile only in patient room, immobile.

### Statistics

Continuous data were presented as mean value ± standard deviation, categorical data as absolute and relative frequency. PSC and the functional outcome were the two binary endpoints. Age (in years and in tertiles), Garden classification (degree of displacement I–IV), preoperative period (in tertiles), ASA score (<3 vs 3), and postoperative weight-bearing (complete relief vs partial vs full weight-bearing) were examined as potential factors. The effect of potential factors on endpoints was estimated with a multivariable logistic regression analysis and 95% confidence intervals were calculated. The null hypothesis Odds Ratio (OR) = 1 was checked by the Wald test. The likelihood ratio test was used to test for deviation from linearity. If the result was significant, the risk of the factor on the outcome parameters examined was determined using the odds ratio. Data were analyzed using Microsoft Excel and Stata 14 (Stata Corp., College Station, TX). A two-sided p-value was determined to be statistically relevant. In addition to the multivariate analysis we made an univariate analysis of factor patient age, divided in two groups of younger and elderly patients, using the chi-square test with a significance level of p = 0.05. This calculation was performed using IBM SPSS Statistics 19 for Windows.

### Ethics approval and consent to participate

All experiments were performed in accordance with applicable guidelines and regulations. The study was conducted anonymously. It is impossible to assign one of the included cases to a person now. Nevertheless, patients or family members provided written informed consent. The Ethical committee of the Medical Council of Brandenburg, Germany (Landesärztekammer Brandenburg) of approved the experimental protocol (No. S 16(a)/2019).

## Results

### Patient collective and surgeons

The mean patient age was: 72 years (<36 min, 96 max). 25 of 56 patients (44,5%) were male. Further patient data is shown in Table 1. The operations were performed by 21 different experienced trauma surgeons or trainees under the supervision of experienced trauma surgeons. In each case, the surgeons used three screws (Asnis III Cannulated Screw System Stryker 8.0 mm) with partial thread. There was no difference in screw configuration with a caudal screw and two other screws above. The screw positioning was correct in 98% of the included cases. In one case the tip apex distance was too short.

**Table 1:** Description of the cohort.

Description of the cohort	
Age	Mean age: 72 years (<36 min, 96 max), median value: 78.5 years, standard deviation: ±17 years
Sex	male: 25 (44,5%), female: 31 (55,5%)
Garden	Garden I: 73%, Garden II: 16%, Garden III: 11%
Pauwels	Pauwels I: 73%, Pauwels II: 21%, Pauwels III: 5%
AO Classification	31-B1: 73%, 31-B2: 27%, 31-B3: 0%
ASA	ASA 1: 9%, ASA 2: 29%, ASA 3: 62%
Walking distance	unlimited: 45%, up to 500 m: 21%, up to 50 m: 16%, mobile only in the patient room: 11%, immobile: 7%

### Classification of femoral neck fractures

The 56 included femoral neck fractures were classified according to Garden, Pauwels and AO classification. In 73% of the cases a Garden I fracture was present, in 16% a Garden II, in 11% a Garden III^15^. In 73% of the cases a Pauwels I fracture was present, in 21% a Pauwels II, in 5% a Pauwels III^16^. In 73% of the cases a 31-B1 fracture was present, in 27% a 31-B2 and in 0% a 31-B3, according to AO Classification.

### Distribution of displacement of femoral neck fractures

The femoral neck fractures were undisplaced in 93% of cases in the elderly age group (≥80 y.o.) and in 57% of cases in the younger age group (<80 y.o.) (chi-square = 9.0, DF = 1, p = 0.01). Table 2 shows the distribution of displacement among femoral neck fractures.

**Table 2:** Distribution of displacement of femoral neck fractures among the two age groups.

	Femoral neck fractures, N (%)		
	Displaced	Undisplaced	Total
Younger age group (<80 y.o.)	13 (43%)	17 (57%)	30 (100%)
Elderly age group (≥80 y.o.)	2 (7%)	24 (93%)	26 (100%)

### Preoperative and intraoperative time

The preoperative period was defined as the period from the arrival of the patient in the emergency room to the incision in the operating room. The average preoperative period was 791 min. (standard deviation ± 859), the median value: 417.5. The maximum preoperative period lasted 4412 min., the minimum 101 min. Of the 56 included cases 2 operations were delayed by more than 24 hours and 4 were delayed by more than 48 hours. The mean intraoperative time was 35 min. (standard deviation ± 9), the median value: 34.5. The maximum intraoperative time lasted 55 min., the minimum 21 min.

### Reduction of femoral neck fractures and screw positioning

In 73% of the included cases, reduction was not required as the fractures were undisplaced. Each displaced femoral neck fracture was reduced. Thus, in 27% of the included cases, reduction of the femoral neck was performed. In one case of the 15 reduced fractures the reduction was insufficient.

### Postoperative weight-bearing

There was no unified postoperative weight-bearing protocol in the department. Postoperatively, in 7% of cases weight bearing was not allowed, in 82% partial weight-bearing (sole contact of the operated leg at mobilization) and in 11% full weight-bearing (full contact of the operated leg treated with the ground during mobilization). It seemed that the decision about weight bearing was made arbitrarily by the ward doctor.

### Procedure – specific complication and functional outcome

In 23 (41%) of the 56 included cases a PSC developed within 12 months after the fracute event with subsequent femoral neck cannulated screw fixation. In 34 (61%) of the 56 included cases, the functional outcome 12 months after the fracture event with subsequent femoral neck cannulated screw fixation was worse than pre-traumatic. In the remaining 22 cases (39%), the pre-traumatic functionality was restored. Table 3 shows the distribution of the PSC after femoral neck cannulated screw fixation.

**Table 3:** Tabular representation of the individual procedure-specific complications for displaced and undisplaced femoral neck fractures (Note! In the table, several procedure-specific complications apply to fewer patient cases - duplication).

Procedure-specific complication and reoperation	n/56
Reoperation	10 (17,9%)
Femoral head necrosis	2 (3,6%)
Infection	0 (0%)
Nonunion	2 (3,6%)
Femoral neck shortening	13 (23,2%)
Screw loosening	11 (19,6%)
Implant penetration	5 (8,9%)

### Influence of the factors on the procedure - specific complication and the functional outcome

**Figure 1:**
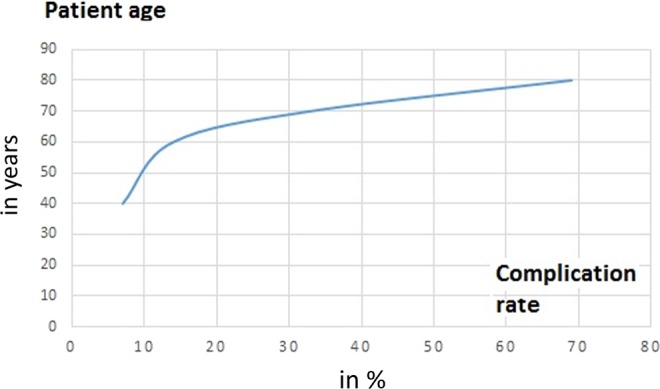
Graphical representation of the distribution of the rate for PSC as a function of the patient’s age.

Factor increasing patient age showed a statistically significant influence on the outcome parameter PSC (OR 1.09 (95% confidence interval 1.02–1.16), p = 0.01). Figure 1 shows a graphic representation of the distribution of the rate for a PSC according to the patient age. In our cohort, 69% (n = 18) of patients from age 80 onwards suffered a PSC. In the age group of 70–79 years, the rate for a PSC was about the average (41%) of the cohort for a PSC. None of the remaining factors examined showed a statistically significant influence on both outcome parameters PSC and functional outcome. The results are shown in Tables 4 and 5. The influence of the factor postoperative weight-bearing on the functional outcome could not be calculated, because most of the patients were treated postoperative with partial weight-bearing.

**Table 4:** Tabular representation of the influence of the factors on the procedure-specific complication.

Procedure-specific complication	Odds Ratio (95% confidence interval)	p-value
Age	1,09 (1,02 to 1,16)	0,01
Garden	1,07 (0,30 to 3,72)	0,91
Preoperative period	0,99 (0,46 to 2,17)	0,99
ASA	0,65 (0,13 to 3,19)	0,60
Postoperative weight-bearing	6,06 (0,50 to 73,9)	0,16

**Table 5:** Tabular representation of the influence of the factors on the functional outcome.

Functional Outcome	Odds Ratio (95% confidence interval)	p-value
Age	0,97 (0,92 to 1,01)	0,17
Garden	1,25 (0,43 to 3,60)	0,68
Preoperative period	0,69 (0,33 to 1,45)	0,33
ASA	0,87 (0,22 to 3,42)	0,84
Postoperative weight-bearing	N/A	N/A

### Influence of factor increasing patient age on the procedure - specific complication and the functional outcome

Our univariate analysis of factor patient age, divided in two groups of younger (<80 y.o.) and elderly patients (≥80 y.o.), showed highly statistically significant results for both PSC (chi-square = 15.9, DF = 1, p = 0.01) and functional outcome (chi-square = 5.35, DF = 1, p = 0.02). The elderly age group suffered a PSC in 69% (n = 18) of cases and the younger group in 17% (n = 5) of cases. The elderly age group suffered a worse functional outcome in 77% (n = 20) of cases and the younger group in 47% (n = 14) of cases.

## Discussion

In our study with 56 cases of displaced and undisplaced femoral neck fractures treated by cannulated screw fixation with a 12 month postoperative observation period for each patient, 41% (n = 23) of the patients developed a PSC and 61% (n = 34) of the patients 12 months after the fracture event had a poorer functional outcome compared to pre-traumatic functionality. We were able to identify the patient age as an influencing factor on the outcome parameter “PSC”. An influence of the other investigated factors (Garden classification, preoperative period, ASA and postoperative weight-bearing) on the PSC was not found. None of the factors had any influence on the functional outcome.

### Procedure – specific complication

A slightly higher complication rate in the present study (41%) is found, when comparing the results with the literature (32.3–38.8%)^3–7^. Table 6 shows a detailed description of this comparison. The results for a femoral head necrosis were between 5.2–9.5% in the literature compared to 3.6% in the present study, for a nonunion between 3.1–24.3% in the literature compared to 3.6% in the present study, for a femoral neck shortening 15.9% literature compared to 23.2% in the present study and for a reoperation between 8–27.6% in the literature compared to 17.9% in the present study. When comparing the results of the present study with the literature, it must be taken into account that there are differences in the methods of the compared studies. This concerns for example the postoperative mobilization of the patients, the implant material used, the screw positioning and the hospital stay. Furthermore, in the meta-analysis of Pitzl and the study of Chen, there were examined displaced and undisplaced femoral neck fractures treated by head-preserving operative methods (dynamic hip screw and cannulated screws)^5,7^. In the meta-analysis of Oñativia, there were examined undisplaced femoral neck fractures treated by cannulated screw fixation^6^. In the study of Wang, there were examined displaced femoral neck fractures treated by head-preserving operative methods (dynamic hip screw or cannulated screws)^3^. In the study of Manohara, there were examined undisplaced femoral neck fractures treated by cannulated screw fixation^14^.

**Table 6:** Tabular representation of procedure-specific complications compared to literature.

Complication and reoperation	present study, n = 56	Pitzl M (2007), n = 4500 (meta-analysis)	Chen C (2017), n = 86	Oñativia IJ (2018), n = 1568 (meta-analysis)	Wang Z (2018), n = 180	Ruben M (2014), n = 100
Osteosynthesis failure	41%	38,8%			32,3%	
Femoral head necrosis	3,6%	9,5%	7,1%			5,2%
Infection	0%	1%				
Nonunion	3,6%	24,3%	4,5%		23,5%	3,1%
Femoral neck shortening	23,2%		15,9%			
Screw loosening	19,6%		22,7%			
Implant penetration	8,9%	7,8%				
Reoperation	17,9%	27,6%		8–16%		8,3%

### Factor patient age

The patient age was identified as an influencing factor on PSC. We stated that the risk of PSC with rising patient age increased by 9% per year within the investigated cohort. The rate for PSC was 69% in the elderly patient group and 17% in the younger group. Within the age group of 70–79 years, the rate for PSC was about the average (41%) of the cohort. With an above average complication rate in the elderly patient group, we believe that at the latest from an age of 80 years cannulated screw treatment of femoral neck fractures should be avoided and an alternative procedure should be considered.

There are several reasons to explain the role of rising patient age for PSC. Higher age is known to favor the development of osteoporosis. The failure of bone tissue in osteoporotic bones leads most often to osteosynthesis failure^17–19^. Further studies have shown that holding power of internal fixator screws decreases by 1000 Newton (or 50%) per 1 mm loss of cortical bone width compared to a healthy cortex^20,21^. The worsening of blood flow to the femur head and the impossibility of compliance with partial weight bearing can be considered as other reasons for osteosynthesis failure in elderly patients.

Some studies, limited to undisplaced femoral neck fractures, have already shown a statistically significant influence of patient age on osteosynthesis failure and other complications^8–10^. In a study on femoral neck fractures treated by head-preserving operative methods, an influence of the following factors could be found on revision risk: the female sex (OR 1.79), increased BMI for a 5 point increase (OR 1.19), displaced fracture (OR 2.16), unacceptable implant position (OR 2.70)^22^. However, an association with age could not be determined. When comparing the results with this study, it should be noted that our study included only femoral neck fractures treated by cannulated screw fixation, possibly explaining the different results of both studies. The comparison of our results with another study on femoral neck fractures treated by head preserving operative methods was interesting^14^. An association between patient age and complication rate was ruled out. However, there were only undisplaced femoral neck fractures included.

### Distribution of displacement of femoral neck fractures

We calculated the distribution of displacement of femoral neck fractures among the two age groups. The younger age group showed a significantly higher proportion of included cases with displaced femoral neck fractures than the elderly age group. Most likely, elderly patients with displaced femoral neck fractures were treated more frequently with a different surgical procedure (e.g. hip replacement) in accordance with recommendations of known literature^23–25^. Therefore, these cases were not included in the study on cannulated screw fixation. The choice of surgical procedure by trauma surgeons led to a pre-selection of femoral neck fractures with regard to their displacement. Even with a more advantageous pre-selection, the elderly age group showed a worse outcome.

### Limitations

The limitations of the study are: it is a retrospective data analysis with a moderate case number of 56 included cases, in which the functional outcome was obtained by a phone call with the further treatment physician. With this study power generalization is difficult. Thus, the present study can serve as a supplement to the current literature. Furthermore, factors such as e.g. osteoporosis, which were not examined in this study, might affect the results. The influence of the factor postoperative weight-bearing on the functional outcome could not be calculated. We did not examine for influence on the results due to operative skills and the number of different trauma surgeons. Further studies are necessary to check on the results and to identify the exact high-risk patient age for the cannulated screw fixation.

## Conclusion

A rate of 41% PSC as an outcome parameter in trauma surgery shows a necessity for improvement. The increasing risk of PSC for patients with a femoral neck fracture treated by cannulated screw fixation is associated with rising patient age. A more stable head-perserving operative method or an endoprosthetic procedure should be considered in high-risk patients (≥80 y.o.).

## Data Availability

The data used to support the findings of this study are available from the corresponding author upon request.
